# Electrophysiological and Neuropsychological Predictors of Conversion to Schizophrenia in At-Risk Subjects

**DOI:** 10.3389/fnbeh.2013.00148

**Published:** 2013-10-21

**Authors:** Tomiki Sumiyoshi, Tomohiro Miyanishi, Tomonori Seo, Yuko Higuchi

**Affiliations:** ^1^National Center of Neurology and Psychiatry, Kodaira, Tokyo, Japan; ^2^Department of Neuropsychiatry, University of Toyama Graduate School of Medicine and Pharmaceutical Sciences, Toyama, Japan

**Keywords:** event-related potentials, mismatch negativity, dMMN, schizophrenia, cognition, early intervention

## Abstract

Patients with schizophrenia show neurophysiological and psychological disturbances before the onset of the illness. Mismatch negativity (MMN), an event-related potential, has been shown to be associated with cognitive function. Specifically, duration MMN (dMMN) amplitudes have been indicated to predict progression to overt schizophrenia in subjects with at-risk mental state. The aim of this article is to provide a hypothesis that a combined assessment of dMMN and neuropsychological performance would enhance accuracy for predicting conversion to schizophrenia in at-risk subjects. Data from these neurocognitive modalities in subjects with first-episode schizophrenia (FES) are also presented. There is accumulated evidence that converters to schizophrenia among at-risk subjects show significantly smaller dMMN amplitudes than those in healthy control (HC) subjects at the frontal lead before the onset. In fact, the amplitudes in these converters have been reported to be similar to those in FES to begin with. dMMN current source density, by means of low-resolution brain electromagnetic tomography, was significantly lower in FES than HC subjects, especially in some medial temporal regions which are implicated in the pathophysiology of schizophrenia. Importantly, dMMN current density in the frontal lobe was positively correlated with working memory performance in FES subjects. These findings indicate the utility of the combination of electrophysiological/neuropsychological assessments for early intervention into patients with schizophrenia and high-risk people.

## Introduction

Shorter duration of untreated psychosis (DUP) has been associated with better prognosis in schizophrenia (Jackson and McGorry, 2009). Also, early intervention into individuals who are at risk of developing psychosis is important to attain better long-term outcome (Jackson and McGorry, 2009). There is a suggestion that brain-related markers, such as subtle morphological changes revealed by magnetic resonance imaging, may provide a tool to identify at-risk people vulnerable to schizophrenia (Takahashi et al., 2009). Accordingly, we reported the utility of electrophysiological measures, such as event-related potentials (ERPs), as a sensitive and feasible biomarker for the detection of individuals who later developed schizophrenia (Higuchi et al., 2013b) and early intervention into the illness (Higuchi et al., 2013a).

In this paper, we provide a theory for electrophysiological and neuropsychological predictors of outcome in early psychosis. The topics include: (1) cognitive function in prodromal phase psychosis, as measured by neuropsychological performance; (2) the role for mismatch negativity (MMN), a component of ERPs, in early detection of schizophrenia; and (3) three-dimensional current source imaging of MMN and its relation with cognitive performance in early schizophrenia.

## The Psychosis High-Risk State

The concept of the psychosis high-risk state has been reported in several ways (e.g., Fusar-Poli et al., 2013). Starting treatment in the early phase of psychosis, or minimizing DUP, is important to improve long-term outcome for patients. If we can start intervention in the prodromal phase, it may prevent progression to psychosis. For this purpose, there have been efforts to establish biological or neuropsychological markers to identify high-risk people who are likely to develop schizophrenia later, which is the main focus of this article.

## Cognitive Function Based on Neuropsychological Measures

There is abundant evidence that cognitive function is impaired in patients with schizophrenia. Usually, the deficit is measured by neuropsychological test batteries, such as the MATRICS Comprehensive Cognitive Battery (Green et al., 2004; Nuechterlein and Green, 2006). In schizophrenia and related psychoses, several domains of cognitive function are disturbed with a 1–2 standard deviation decline. The cognitive deficit has been reported to provide a vulnerability marker of schizophrenia, so one would expect similar disturbances in high-risk people for the disease. In fact, a recent meta-analysis of cognitive functioning in people at risk for psychosis indicates impairments in almost all cognitive domains which are typically affected in schizophrenia, i.e., executive function, verbal fluency, attention, visual memory, verbal memory, working memory, and social cognition, with a milder degree (Fusar-Poli et al., 2012).

**Figure 1:**
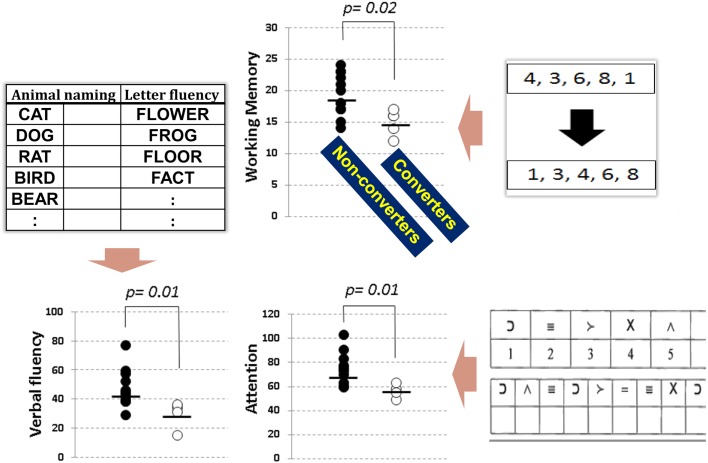
**Neuropsychological performance, as evaluated by the BACS, in subjects with at-risk mental state**. Transition to overt schizophrenia was predicted by working memory (top), verbal fluency (bottom left), and attention (bottom right) before the onset of illness (extracted from Higuchi et al., 2013b).

The Brief Assessment of Cognition in Schizophrenia (BACS) battery (Keefe et al., 2004) is one of the most frequently used tests to evaluate cognitive impairment of schizophrenia in Japan. It takes only approximately 30 min to complete, and covers key cognitive domains specifically impaired in schizophrenia (Keefe et al., 2004; Kaneda et al., 2007). We recently investigated performance on the BACS in people with at-risk mental state (ARMS), and compared baseline data between subjects who later developed schizophrenia and those who did not (Higuchi et al., 2013b). As demonstrated in Figure 1, the two groups performed differently in working memory, verbal fluency, and attention. These results are generally consistent with the literature (Fusar-Poli et al., 2012), and indicate impairment of frontal lobe function in vulnerable people plays a role in the progression to schizophrenia (Higuchi et al., 2013b; Miyanishi et al., 2013). Specifically, a recent meta-analytic study (De Herdt et al., 2013) reports worse working memory and visual (learning) memory for converters compared to non-converters, supporting the above concept based on results from a larger number of subjects.

## Mismatch Negativity

As discussed, data from neuropsychological performance may provide some information to identify high-risk individuals who later develop psychosis. However, the sensitivity of neuropsychological evaluation to predict conversion to schizophrenia may be less than that of negative symptoms (Riecher-Rossler et al., 2009). This prompts the search for neurocognitive markers from other modalities, such as ERPs and other electrophysiological paradigms.

MMN is a pre-attentive component of ERPs. When auditory cortex automatically detects a change of stimuli, attention shifting occurs in frontal cortex (Jahshan et al., 2012a,b). This neural process generates MMN. For the measurement of MMN, auditory stimuli were delivered to subjects. Standard and target tones with different durations were randomly presented in the case for duration MMN (dMMN). During the measurement, subjects are requested to pay attention to a silent animation movie and ignore the tones. MMN is obtained by subtracting standard waveforms from target waveforms.

**Figure 2:**
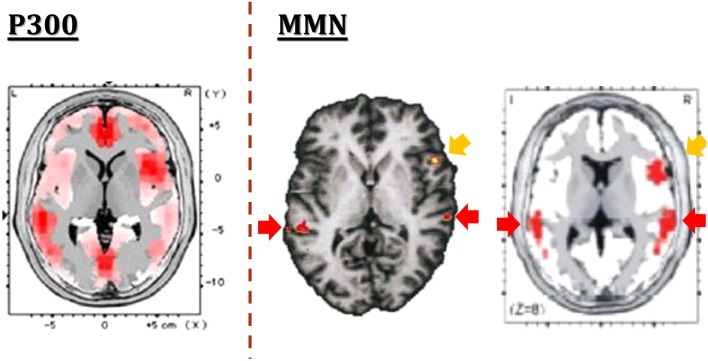
**The generators of ERPs**. In contrast to multiple generators for P300 (left), putative generators for MMN are limited to superior temporal gyrus and prefrontal cortex, as demonstrated by fMRI (center) and EEG-LORETA (right) methods. Images are extracted from Higuchi et al. (2008) (left), Opitz et al. (2002) (center), and Marco-Pallares et al. (2005) (right), respectively.

One of the strength of MMN is the limited number of generators, in contrast to the case for P300, another component of ERPs (Figure 2). The generators for MMN are assumed to be located mainly on superior temporal gyrus and prefrontal cortex. This facilitates functional imaging evaluation. Importantly, MMN amplitudes have been shown to be decreased in schizophrenia with a large effect size (Umbricht and Krljes, 2005). Specifically, dMMN amplitudes have been found to be decreased also in ARMS subjects (e.g., Jahshan et al., 2012a).

**Figure 3:**
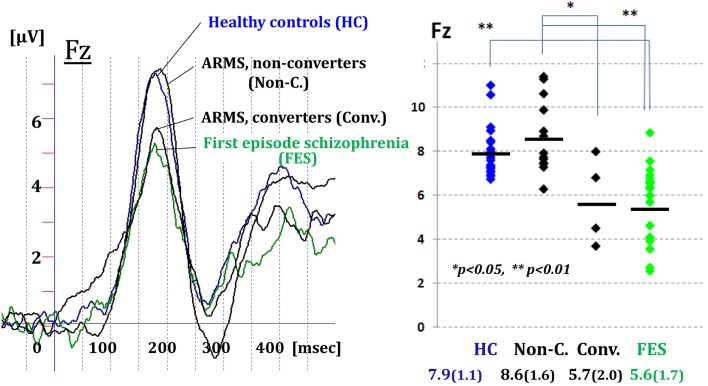
**Prediction of transition to schizophrenia by dMMN in ultra-high risk subjects**. MMN waveforms at the frontal lead are shown for healthy controls, ARMS subjects, and first-episode schizophrenia. Converter subjects showed reduction in the amplitudes before the onset, similar to patients with first-episode schizophrenia. By contrast, MMN amplitudes of non-converters resembled those of healthy controls (Higuchi et al., 2013b).

Figure 3 demonstrated MMN waveforms at the frontal lead for healthy controls (HCs), ARMS subjects, and first-episode schizophrenia (FES). Converter subjects showed reduction in the amplitudes before the onset, similar to patients with FES. By contrast, MMN amplitudes of non-converters resembled those of HCs (Higuchi et al., 2013b). These results are consistent with some recent reports from other groups of investigators (Bodatsch et al., 2011; Atkinson et al., 2012; Jahshan et al., 2012a; Shaikh et al., 2012). A novel finding in our study was a positive correlation between MMN amplitudes and verbal fluency in ARMS subjects (Higuchi et al., 2013b). This indicates word production during a given time would provide an estimate of an electrophysiological activity which is predictive of progression to overt schizophrenia.

## Other Electrophysiological and Neuropsychological Biomarkers

There is evidence that amplitudes of P300, another component of ERPs reflecting attentive cognitive abilities, are reduced in at-risk subjects (e.g., Nagai et al., 2013). As noted above, visual memory has been reported to differentiate between converters and non-converters in individuals vulnerable to developing schizophrenia (De Herdt et al., 2013). Further efforts are required to refine the use of these biomarkers for early detection of psychosis.

## Three-Dimensional Imaging of dMMN Current Density

Localization of generators for ERPs provides valuable information. For this purpose, the low-resolution brain electromagnetic tomography (LORETA) methods have been used (Pascual-Marqui, 1999, 2002). In these analyses, current source density of electrical activity is calculated from scalp EEG. Specifically, the LORETA methods can perform voxel-by-voxel comparisons of current source density.

Recently, researchers from the University of California San Diego conducted three-dimensional imaging of dMMN current density in control subjects and patients with chronic schizophrenia (Takahashi et al., 2012). In that study, the mean duration of illness was 24 years, which was lengthy. The comparison between the two groups indicates reduced activations in the cingulate gyrus and medial frontal gyrus in patients (Takahashi et al., 2012).

**Figure 4:**
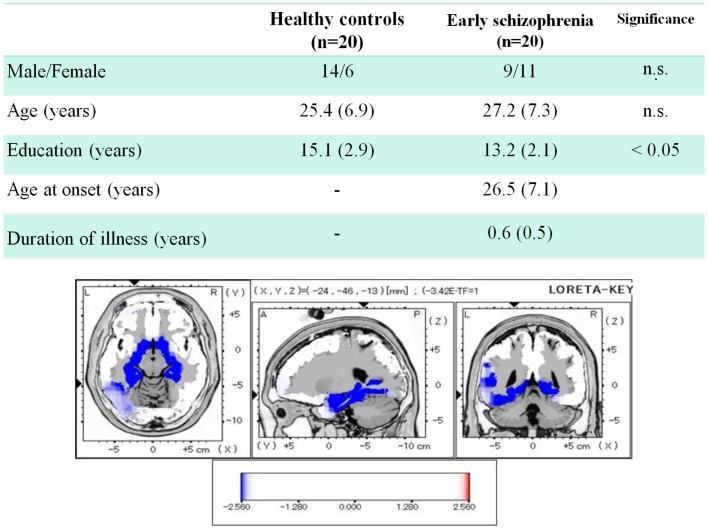
**Three-dimensional imaging of dMMN current density in *early* schizophrenia**. Duration of illness was <1 year for all patients. Comparison between healthy subjects and patients showed decreased current density in such brain regions as bilateral parahippocampal gyrus, left fusiform gyrus, right hippocampus, and left anterior cingulate gyrus (data extracted from Miyanishi et al., 2013).

Regarding the *early* phase of schizophrenia, we recently reported data from patients whose mean duration of illness was <1 year (Miyanishi et al., 2013). Figure 4 demonstrates the comparison of dMMN current density between healthy subjects and patients. Early schizophrenia patients showed decreased current density in medial temporal lobe structures and anterior cingulate gyrus, i.e., brain areas related to the pathophysiology of schizophrenia (Jensen et al., 2004; Hao et al., 2009).

**Figure 5:**
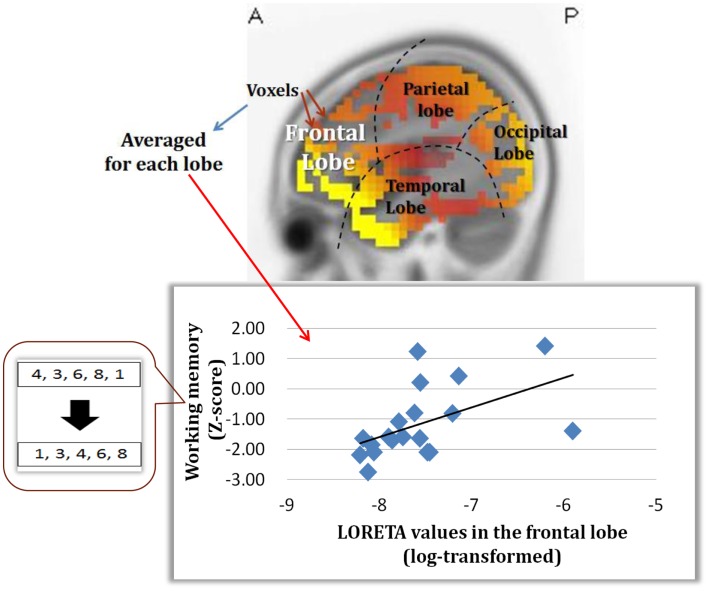
**Correlation between dMMN current density in the frontal lobe and working memory, as evaluated by the BACS-J, in patients with *early* schizophrenia**. (Miyanishi et al., 2013).

An important part of our study was to determine if the change in dMMN activations is associated with neuropsychological performance. As demonstrated in Figure 5, dMMN current density in the frontal lobe is positively correlated with working memory, as measured by the BACS, in early schizophrenia patients (Miyanishi et al., 2013). These findings are consistent with the concept that the prefrontal cortex plays a major role in this cognitive domain. Further study is warranted to see if the association between dMMN current density in the frontal lobe and working memory is specific to schizophrenia, but not HCs, and if dMMN current density predicts progression to schizophrenia in at-risk people.

## Conclusion

This paper provided a hypothesis regarding a role for neuropsychological and electrophysiological markers in intervention into early psychosis and high-risk subjects. The combination of these modalities of neurocognition would be expected to facilitate early detection of subjects who are likely to develop psychosis, and identification of those who need immediate treatment.

## Conflict of Interest Statement

The authors declare that the research was conducted in the absence of any commercial or financial relationships that could be construed as a potential conflict of interest.
